# Teaching science and mathematics to students with visual impairments: Reflections of a visually impaired technician

**DOI:** 10.4102/ajod.v4i1.194

**Published:** 2015-11-04

**Authors:** Mbulaheni Maguvhe

**Affiliations:** 1 Department of Inclusive Education, University of South Africa, South Africa

## Abstract

This study reports on factors that limit the participation of blind and partially sighted learners in mathematics and science education. Since the teacher, still remains one of the most crucial factors in any education system, the researcher deemed it important to investigate the role of the teacher as understood by a blind technician in promoting the participation of blind and partially sighted learners in mathematics and science subjects, which few of these learners take beyond primary school. A case study was conducted interrogating a blind technician, who regards himself as an unqualified scientist, in his understanding of various school factors that could entice blind and partially sighted learners to participate in mathematics and science education, and to promote their retention in related professions. The participant thus drew from his own experiences of the school environment and wider concentric social institutions. A semi-structured interview schedule was followed and the responses were recorded by mutual consent. Analysis was conducted based on questions put to the participant. The study revealed that teacher motivation and mentorship in mathematics and science methodologies and the use of tools for learner empowerment are lacking. It further revealed that teachers lack the requisite skills in special education to harness learner potential in mathematics and science. This situation necessitates government action in teacher training and development.

## Introduction

This research study was in the form of a case study. The study drew on the experience of one blind male technician. The technician has great ambitions in the pursuit of science and technology, but with little support. The aim of the study was to understand the teaching and learning playing field. The focus was on what could be done to increase the involvement of blind and partially sighted learners in mathematics and science education. Of the 22 registered schools for blind and partially sighted learners, only two (Prinshof in Gauteng and Pioneer in Western Cape) offer science and mathematics to blind and partially sighted learners. This means that approximately 600 learners out of 3000 learners (learners that these schools together could accommodate) benefit from science and mathematics subjects. The participant considers teachers as the primary resource that ought to be equipped with knowledge, skills, attitudes and values to enable them to inspire and stimulate learners’ interest in the sciences. Given the fact that teachers of blind and partially sighted learners play multiple roles in shaping the lives of learners (Spungin & Ferrell 2007), teachers should be the focus of empowerment (Carl 1995) so that they could in turn empower their learners.

This study has wide application within and beyond South Africa’s borders. However, problems with teaching mathematics and science for the blind and partially sighted learners are also experienced elsewhere (Design Science 2011; Texas School for the Blind and Visually Impaired 2007). Sahin and Yorek (2009) support the previous argument when they assert that learners have regarded science as a difficult subject because of difficult and abstract concepts. They continued arguing that science teaching has been dependent mostly on visual instruction. This makes it difficult for partially sighted learners included in a regular classroom to learn the concepts. Blind learners on the other hand, have no visual input at all. They need to learn using the other senses such as touching and hearing.

The fact that this study was conducted in South Africa is of potential benefit to researchers and teachers, particularly in Africa where science and mathematics subjects would play a crucial role in industrial development. South Africa is the largest economy in Africa. It still provides resources for research and development, which makes the production and distribution of knowledge comparatively cheaper and the audience for this study larger than the national platform. This study could be of value to education practitioners in other countries across the globe as well, since mathematics and science education for the blind and partially sighted learners still requires practitioners from different parts of the world to share ideas on best practices.

### Literature review

While science and mathematics education is easily accessible to fully sighted children, it is less accessible to blind and partially sighted children since many of its concepts are presented graphically, and there are many concepts that cannot be explored by touch and are put across through visual observation (Design Science 2011; Kalra *et al.* 2009; Maguvhe 2005; Sahin & Yorek 2009). For several reasons, the chances are slim for blind and partially sighted children to pursue a science education (Schleppenbach 1996). Many people fear sight loss more than they do other impairments (Dickerson, Smith & Moore 1997) and tend to think that without sight, one would not be able to do any of the most taxing mental activities that sighted people are typically able to do. The connotation being that blindness overlaps with limited capacity (Maguvhe 2003:118).

The fact that blindness is generally the most dreaded disability worldwide invokes societal sanctioning for those belonging to this group (Dickerson *et al.* 1997). This is to the extent that human attitudes compound the negative effects of physical barriers to the education of blind and partially sighted learners (Fraser & Maguvhe 2008). The absence of accessible media for the presentation of concepts in fields of study with a large amount of complex visual information - such as mathematics, engineering, physics, chemistry and biology - has traditionally been highlighted as a legitimate reason to conceal these areas of learning from large-scale entry and pursuance by the blind and partially sighted learners (Cryer 2013; Schleppenbach 1996).

In cases where apparatus have been modified for use by blind and partially sighted learners, and information presented in accessible formats, this group of learners have been observed to perform competitively with their typically sighted counterparts (Sahin & Yorek 2009). Hence, the main reason why blind and partially sighted learners have been unable to participate in pure sciences in large numbers is more about the lack of appropriate access technologies and teacher attitudes than about the psychological incapacity of blind and partially sighted learners themselves.

Owing to a combination of attitudinal barriers to the participation of blind and partially sighted learners in activities that are significant to society, and society’s own limitations in creating technologies that enable blind and partially sighted learners to interface with scientific information, there has been a paucity of blind and partially sighted learners entering and excelling in the fields of science and mathematics. Consequently, there are few blind and partially sighted mentors and role models to encourage members of the blindness sector to pursue their studies in the hard sciences as professional niches (Schleppenbach 1996).

The literacy rate among the blind and partially sighted in developing countries is below 3% (Kalra *et al.* 2009). Since children with visual impairments have marginal chances of starting school and going through basic education, it is critical to appreciate that the actual number of the blind and partially sighted pursuing the hard sciences in developing countries is small, given the general paucity of enabling resources for access to information.

An even gloomier picture emerges of the proportion of blind and partially sighted learners who pursue science and mathematics as lifelong professions. Most of these learners do not take mathematics and science subjects mostly because their teachers give them the impression that these subjects are inaccessible to learners with sight problems. The underlying problem is that often the teachers themselves do not have adequate direct experience with teaching blind and partially sighted learners (Sahin & Yorek 2009; Stefanich & Norman 1996:51).

Consequently, many teachers find it difficult to help their learners with appropriate knowledge acquisition strategies for science and mathematical subjects. Through the attitudes of teachers, many schools for the blind and partially sighted learners have no conviction that their own learners are good enough to do well in the pure sciences. However, the fact remains that blind and partially sighted learners are endowed with the same cognitive capacity as typically sighted learners (Kumar, Ramasamy & Stefanich 2001). Those schools therefore do not offer science and mathematics to their entire student body. Because of a combination of attitudinal and technological limitations in schools, few blind and partially sighted learners become physical and biological scientists.

According to Maguvhe (2005), many of these learners who are from schools for the blind and partially sighted tend to affirm mainstream attitudes, while smaller numbers think independently and challenge prevailing school expectations. Survivalist attitudes are important for every learner and quintessential to the success of blind and partially sighted learners if they aspire to overcome the social factors that militate against their educational pursuits. According to Education White Paper 6, special schools will be used as resource centres for inclusive education (Department of Education 2001). Such schools therefore ought to be in a position to act as examples - even in the degree and variety of equipment they stock and the subjects they offer to their learners.

The South African government recognises science, mathematics and several other professions that are dependent on these subjects (such as medicine) as scarce and critical skills. Jobs that apply those skills must also be accessible to blind and partially sighted learners. To achieve the quota of people with disabilities in different professional fields, especially in fields considered scarce and critical (fields that matter most for technological development), there needs to be a firm resolve to educate learners with disabilities in those fields. This research focused specifically on the involvement of blind and partially sighted learners in these core fields of study.

Schleppenbach (1996) suggests two areas of learner learning that need to be covered when teaching blind and partially sighted learners - their educational and technological needs. Blind and partially sighted learners must from time to time be consulted in order to determine ways of accommodating them in line with their suggestions on their academic and technological needs. Hence, the area of learning one finds difficult is not isolated from the kind of technological assistance the learner deems helpful. The mentor’s inputs also contribute to the formation of the ultimate programme to be used.

## Research questions

The researcher used the following question to collect data: How does a blind technician experience science and mathematics education in a special school?

## Methodology

### Case study research

A case study is a detailed study of ‘a single entry’ (McMillan & Schumacher 2010:344), such as ‘an activity, event, process or individuals’ (Creswell 2008:476; David & Sutton 2011:165). Thus, case studies characteristically collect large quantities of data to enable the researcher to develop patterns of thought, which ultimately produce meaningful information on single cases. Although findings from case studies are derived from in-depth analysis of phenomena, they are not generalisable because they examine a limited number of phenomena with characteristics of interest to the researcher. However, insights from case studies lead to wider studies, which are more generalisable. The current study is a ‘case-focused study’ of an individual with unique characteristics of interest to the researcher (McMillan & Schumacher 2010:345).

### Background information on the participant

The participant who gave inputs in this study is a totally blind, 35-year-old man who holds a matric certificate for his formal education. He has 13 years’ working experience. He lacks the ability to use visual stimuli to integrate environmental information meaningfully. Further, the participant uses a white cane to help him navigate his way.

A face-to-face interview was held with only one participant, owing to his unique technical acumen in a science-related field - an area that few blind and partially sighted learners pursue in South Africa. It should be noted that, since this was a small case study, the chance of many teachers and former learners expressing divergent views does exist.

The researcher deemed it necessary to conduct a follow-up telephonic interview to verify the participant’s responses and comments recorded during the face-to-face interview.

Data was presented in thematic sections, which arose from responses received from both the face-to-face interview and the follow-up interview. The data was then qualitatively examined for meaning. The views of the blind technician were sought on the visibility, benefits and challenges of mathematics and science education initiatives in South Africa. The responses of the blind technician were interpreted hermeneutically with reference to documented national policies and published international developments in mathematics and science education for blind and partially sighted learners.

## Results and discussion

It was necessary to identify a blind participant, who had gone through science and mathematics education and succeeded because of his ambition and goal-directed effort, to understand what he considered effective methods and strategies to mediate science and mathematics teaching and learning. The fact that the case study did not solicit information from a teacher was considered revealing because the researcher wished to determine the experiences of ‘a product of teaching’ - not those of the teacher. The researcher felt that an interview with a direct insider in the teaching process might not be the best source of information on the lived experiences of blind and partially sighted learners in subject areas. The researcher believes that conducting similar case studies in different countries could encourage blind and partially sighted readers to use personal interest to meet the challenge of pursuing science and mathematics. Furthermore, conducting similar case studies in other countries could bring to light questions that teachers and policy makers need to answer through appropriate provisioning and prompt information exchanges.

Each of the themes will be discussed below in order to present the results of the interview. Important excerpts from the interviewee’s responses will also be highlighted.

### Theme 1: Accessibility of mathematics and science curriculum

It emerged from this study that the mathematics and science curricula are accessible to the blind and partially sighted learners. This finding was in line with that of Kumar *et al.* (2001) – namely that the blind and partially sighted learners have the same mental capacity to comprehend mathematics and science – as well as Sahin and Yorek’ s (2009) conclusion that blind and partially sighted learners merely need to be appropriately accommodated to enable them to perform as well as their sighted counterparts in those sciences. Varieties of technologies are now available to allow for the participation of blind and partially sighted learners in mathematics and science education.

According to the participant, the main problem is that teachers are not well equipped to teach mathematics and science to blind and partially sighted learners. The participant is of the view that teachers lack practical knowledge of possibilities of blind and partially sighted learners in mathematics and science - hence their doubts about the capacity of their learners in those subjects. They also lack the requisite knowledge of blindness and visual impairment to enable them to provide for the needs of their learners in mathematics and science. Under the present circumstances, learners thrive on their personal ambitions and initiatives. The following verbal quotation illustrates these findings:

‘I believe the curriculum is accessible. There is a lot of technology for braille users today, which includes math and science kits that we can understand. It is the teachers themselves who make it inaccessible. They do not believe in us. This makes them not to go all out giving their best when teaching. Mere knowledge of math or science without knowledge of how to address the needs of the learner is not enough. Oh! Let me tell you a short story. Look, I am blind. Through trial and error, I am now one of the few blind guys who can repair braille machines. When I attended a course for repairing machines in Worcester, I was the only blind person among the sighted. People wondered what I was there for. I said to the trainer ‘*nnakebatla setifikeiti*’ [*I want the certificate. Otherwise I know I can repair machines*]’.

### Theme 2: The state of teacher readiness and preparedness in teaching mathematics and science

The study further revealed that teachers were not well trained to teach the blind and partially sighted learners, and lacked the necessary innovation where resources for the teaching of science and mathematics were limited. This finding concurs with that of Sahin and Yorek (2009), namely that many teachers do not have direct experience in teaching blind and partially sighted learners. They do not know what to do to improve the learning conditions of their learners in cases of marginal resources.

The following verbal quotation illustrates these findings:

‘Today’s teachers, whether they are prepared or not, one cannot tell. It seems as if they are not. Why are they reluctant to teach mathematics and science? They put the blame always on lack of resources. They put the blame on lack of training via workshops. They put the blame on blind and partially sighted learners who cannot understand graphs, maps, tables and so on. Teachers in the olden days, used to make a plan, [*I*] mean improvisation when resources were not enough. They did not always play blame games’.

The response shows that, if teachers make an effort, the blind and partially sighted learners can benefit from mathematics and science education. The participant demonstrated his skills to the researcher by successfully repairing an electric heater and a kettle. The demonstration further illustrated Sahin and Yorek’ s (2009) finding that the blind and partially sighted have equal competencies than the typically sighted, given accessible media for learning. Such findings are very relevant to share with teachers and academics in other countries because they inspire the blind and partially sighted learners to try out their talents and do more in science and mathematics.

### Theme 3: Teacher training and personal development

The study, through opinions expressed by the participant, revealed that teachers lack specialist training to teach blind and partially sighted learners, including rigorously supervised teaching practice during such training. As a result, they were not competent in transferring their knowledge to learners with special learning needs, particularly the blind and partially sighted. These findings are closely related to the observations made by the Catholic Education Office Canberra (2011), namely that teachers are the least confident in undertaking assessment procedures. More recently, Section 27, which advocates for social justice, instructed by the South African National Council for the Blind, reported a dire lack of learner teacher support material (LTSM) and low levels of teacher specialisation in both schools for the blind and partially sighted, as well as full service schools, which they had to provide with consultancy (Hodgson & Khumalo 2015). Spungin and Ferrel (2007) also highlight the multiple roles of a teacher for the blind and partially sighted that are not fulfilled in the current educational terrain in South Africa. By implication, teachers find it difficult to understand their learners through learners’ output. That knowledge gap implies a lack of specialist knowledge, which would otherwise lead to proper diagnosis and informed teaching.

The following verbal quotation illustrates these findings:

‘Like I indicated above, teachers complain about this and that. Well-prepared teachers do not have time to complain. They know they have good skills to teach the blind learners. I think they lack specialist training for teaching the blind learners and just rely on their knowledge of particular subjects, so they have no dedication to their mandate. If their thinking does not change, it will be years and years before we produce well-trained blind mathematicians and scientists’.

Teachers who are not specialists cannot effectively articulate subject matter to blind and partially sighted learners. Sahin and Yorek (2009:19) confirmed similar findings in Turkey, stating that teachers are unable to impart their knowledge of methods, with the result that not all learners are able to participate optimally.

The current result indicates a dire need for training of specialists, a problem that is found in many developing countries. The study is therefore relevant to the needs of other African countries, as it indicates the need for national or regional colleges that train specialist teachers for learners experiencing various barriers to learning. In the Zimbabwean model, teachers go for special education training only after specialising in the teaching of various primary and secondary school subjects – such as science and mathematics for early childhood learners, infants, juniors and secondary school learners - in their mainstream teacher education. Those who choose to proceed to higher diploma studies in special needs education make a choice to specialise in different areas of special education, but they receive generic courses that equip them to meet the needs of learners who have learning needs beyond the teachers’ specialist niches. The results of this study give opportunities for a sub-regional audit of appropriate teacher training models.

### Theme 4: Relevant teacher training programmes

To show the importance of teaching learners different methods of doing things, different authors illustrated how they successfully taught braille users to master scientific and mathematical concepts. Osterhaus (2002) suggested how to set up a mathematics technology corner, explaining methods of teaching graphs to blind and partially sighted learners. Fraser and Maguvhe (2008) illustrated how to use a combination of three-dimensional models to capture the meaning of two-dimensional drawings (such as the structure of a cell). Because many teachers lack the practical skills, they require at least a diploma in special education, with specialisation in blindness and partial sightedness, in addition to their mathematics or science qualifications to enable them to facilitate learning effectively.

The following verbal quotation illustrates these findings:

‘Prof, we need people like you who are also visually impaired like us to advocate on behalf of the helpless [*meaning blind and partially sighted learners*]. You must develop training programmes at your institution for teachers for the blind and partially sighted learners. A hands-on qualification such as a diploma in special education with specialisation in visual impairment would do. Government should appoint people who are well qualified to teach the blind who also specialise in blind matters. Perhaps, if teachers could also be on probation for a long time, they [*government*] may find the right ones’.

While the response points to a good training avenue, the reality is that there is currently no university offering that type of qualification. The University of South Africa used to offer such diplomas, but it stopped. The same university is only planning to offer such diplomas again in the future. The results of this study point to the need for the South African government to look for immediate training solutions - such as sending some teachers to train in special needs education skills, which are in short supply, in other African countries or even further afield.

### Theme 5: In-service training

The study further revealed, through opinions of the blind technician, that mathematics and science teachers need to attend regular staff development workshops covering selected topics in mathematics, science and accommodation suitable for blind and partially sighted learners. The wide publicity of this and similar research will inspire teachers to arrange workshops and seminars with colleagues in other African countries and plan subregional teacher development possibilities. This will enable them to keep abreast of curriculum demands, new developments in their subject areas and emerging technologies for accommodating their learners’ educational and wellness needs.

It also emerged, according to opinions expressed by the participant, that teachers have to develop information-gathering skills through actual research. To that end, they have to create networks with other area specialists the world over. The following verbal quotation illustrates these findings:

‘They [*teachers*] should have workshops for mathematics and science in addition to joining university programmes that equip them to understand the learners they are teaching. They can as well network with colleagues here and abroad and exchange ideas on how to teach blind learners. I mean their [*blind and partially sighted learners*’] abilities and limitations, and how to make a plan to overcome the limitations’.

### Theme 6: Mathematics and science as learning areas for the blind

The present study further revealed that mathematics and science education is essential for the blind and partially sighted. This is important for these individuals’ economic stability and personal physical health. Hence, these subjects are essential, not only for the continued survival of the blind and partially sighted learners, but also for improving their quality of life.

The higher applications of mathematics and science for daily living alluded to by the participant in this case study might be shared by many people who are differently abled throughout the world. This should take into account the fact that mass awareness campaigns on health and street wisdom do not cover all sectors of human populations equally effectively, owing to factors such as limited knowledge of sector-specific communication strategies and the paucity of resources for all-inclusive coverage. The fact remains that a lack of knowledge is perilous to human life. The following verbal quotation illustrates these findings:

‘Yes, Yes, Yes. One cannot live without mathematics and science. Even those who live on the disability grant need to have knowledge of basic mathematics and science for survival. Some of the problems we have with living from hand to mouth arise from a lack of that info. Some of our problems with containing epidemics are due to a multi-generational bankruptcy in science. They need to add, subtract, multiply and divide their money’.

### Theme 7: Blind and partially sighted learners’ mathematics and science setbacks

The study revealed that blind and partially sighted learners find it difficult to pursue mathematics and science subjects because the resources are limited and teachers are not prepared to do their best to resolve the problems they encounter in putting the subject matter across to their learners. It was also revealed that blind and partially sighted learners themselves have no volition to improve their circumstances, because their total learning environments fail to assist them optimally (Fraser & Maguvhe 2008:86). Blind and partially sighted learners need to be proactive to improve their performance in mathematics and science. The following verbal quotation captures the essence of these findings:

‘Yes, lack of resources, unprepared teachers, experiments, graphs, maps and tables are some of the setbacks the blind learners encounter when doing or want to do mathematics and science. However, this should not be an excuse not to teach them’.

## Recommendations

The author of this article recommends that proper support be provided and reasonable accommodation measures be implemented to ensure effective mathematics and science teaching/learning. This sentiment is also echoed by Disability Rights (2015) when it argues that proper teaching and learning would take place if duty bearers take responsibility for exclusion, marginalisation and discrimination in education and are held accountable, and if rights holders would have access to recourse when their rights have been violated. The previous stated source further argues that it is not the responsibility of the parents of blind and partially sighted children to go up and down seeking a door that will open, spending the family’s food budget on transport to move from one school to the next. It is the responsibility of all government officials in the value chain to ensure that blind and partially sighted learners access education through suitable assessment and support by the relevant district education offices. The author of this article further suggests research in the following areas:

1. Would mathematics and science be the preferred options for many blind and partially sighted learners?
2. Would mathematics and science be taught more efficiently and effectively if government could introduce an ongoing teacher training programme to equip teachers with skills in both areas of special needs education that pose barriers to their learners and teaching approaches for particular topics in their subject specialisation?
3. Could mathematics and science be taught more comprehensively with the use of available technologies if principals were familiarised with (and teachers were thoroughly trained in) the use of emerging technologies?

## Conclusion

This case study attempted to determine the factors affecting the participation of blind and partially sighted learners in science and mathematics in schools for the blind. From the findings of the study, it can be concluded that successful mathematics and science education for blind and partially sighted learners requires teacher empowerment through rigorous training and development on both approaches to subject matter and general awareness to the unique learning needs of learners with various degrees of visual loss. It must be noted that, since tSShis was a small case study, the chance of many teachers and former learners expressing divergent views exists, although the findings corroborate the results of similar studies discussed in the literature review. Although case studies are based on such small samples that their results are less generalisable, experiences of people in special populations might have some commonalities. This study could inspire the quest to conduct quantitative studies in several African countries in order to understand the perceptions of former learners as products of the teaching/learning process to determine the needs of current regional education systems.

## References

[CIT0001] Carl A.E., 1995, *Teacher empowerment through curriculum development: Theory into practice*, Juta, Kenwyn.

[CIT0002] Catholic Education Office Canberra, 2011, *Teachers*’ *guide to assessment*, Archdiocese of Canberra and Goulburn, Canberra, viewed 1 February 2015, from http://www.det.act.gov.au/teaching…learning/teachers_guide_to_assessment

[CIT0003] Creswell J.W., 2008, *Research design: Qualitative, quantitative, and mixed methods approaches*, SAGE Publications, London, Thousand Oaks, CA, New Delhi/Singapore.

[CIT0004] Cryer H., 2013, *Teaching STEM subjects to blind and partially sighted students: Literature review and resources (Literature review #6)*, RNIB Centre for Accessible Information, Birmingham, viewed 16 January 2015, from https://www.google.com/url?sa=t&rct=j&q=&esrc=s&source=web&cd=1&ved=0CB0QFjAAahUKEwir4LGQwrPIAhXF7RQKHU1zCGE&url=https%3A%2F%2Fwww.rnib.org.uk%2Fsites%2Fdefault%2Ffiles%2F2013_05_Teaching_STEM.docx&usg=AFQjCNHkZ8nnThoq8TD8ZZZcpWqmj9MPzQ

[CIT0005] David M. & Sutton C.D., 2011, *Social research: An introduction*, SAGE Publications, London, Thousand Oaks, CA, New Delhi/Singapore.

[CIT0006] Department of Education, 2001, *Education white paper 6. Special needs education: Building an inclusive education and training system*, Department of Education, Pretoria.

[CIT0007] Design Science, 2011, *New math to speech technologies to help blind and visually impaired students master mathematics*, viewed 22 June 2015, from https://www.dessci.com/en/company/press/releases/110524.html

[CIT0008] Dickerson L.R., Smith P.B. & Moore J.E., 1997, ‘An overview of blindness and visual impairment’, in Moore J.E., Graves W.H. & Patterson J.B. (eds.), *Foundations of rehabilitation counseling with persons who are blind or visually impaired*, pp. 1–24, American Foundation for the Blind, New York.

[CIT0009] Disability Rights, 2015, email, 20 August.

[CIT0010] Fraser W.J. & Maguvhe M.O., 2008, ‘Teaching life sciences to blind and visually impaired learners’, *Journal of Biological Education* 42(2), 82–89, viewed 01 February 2015, from http://repository.up.ac.za/bitstream/handle/2263/6236/Fraser_Teaching(2008).pdf?sequence=1

[CIT0011] Hodgson T.F. & Khumalo S., 2015, *South Africa: Schools for the blind in* ‘*Shocking*’ *state*, viewed 21 June 2015, from http://mg.co.za/article/201506-11-blind-schooling-in-shocking-state

[CIT0012] Kalra N., Lauwers T., Dewey D., Stepleton T. & Dias M.B., 2009, ‘Design of a braille writing tutor to combat illiteracy’, *Information Systems Frontiers* 11(2), 117–128. http://dx.doi.org/10.1007/s10796-009-9171-2

[CIT0013] Kumar D., Ramasamy R. & Stefanich G., 2001, ‘Science for students with visual impairments: Teaching suggestions and policy implications for secondary educators’, *Electronic Journal of Science Education* 5(3), 1–4.

[CIT0014] Maguvhe M.O., 2003, ‘Being a blind researcher in South Africa: A critical assessment’, *Perspectives in Education* 21(3), 117–119.

[CIT0015] Maguvhe M.O., 2005, ‘A study of inclusive education and its effect on the teaching of biology to learners with visual impairments’, Thesis (Abstract), Department of Curriculum Studies, Faculty of Education, University of Pretoria, Pretoria.

[CIT0016] McMillan J.H. & Schumacher S., 2010, *Research in education: Evidence-based enquiry*, 7th edn., Pearson, Boston.

[CIT0017] Osterhaus S., 2002, *Susan*’*s math technology corner: Teaching a blind student how to graph on a coordinate plane: No tech, low tech, and high tech tools*, viewed 14 January 2015, from http://www.tsbvi.edu/math/graphing.htm

[CIT0018] Sahin M. & Yorek N., 2009, ‘Teaching science to visually impaired students: A small-scale qualitative study’, *US-China Education Review* 6(4), 19–26.

[CIT0019] Schleppenbach D., 1996, ‘Teaching science to the visually impaired: Purdue University’s Visions Lab’, *Information Technology and Disabilities E-Journal* 3(4).

[CIT0020] Spungin S.J. & Ferrell K.A., 2007, ‘Expansion of the role and function of the teacher of students with visual impairments: Providing for students who also have severe multiple disabilities’, position paper for the Council for Exceptional Children (Division of Visual Impairment), viewed 19 January 2015, from http://www.tsbvi.edu/pds/1988-xpansion-of-the-role-of-the-teacher-of-students-with-visual-impairments-providing-for-students-who-also-have-severemultiple-disabilities

[CIT0021] Stefanich G.P. & Norman K.I., 1996, *Teaching science to students with disabilities: Experiences and perception of classroom teachers and science educators*, Special publication of the Association for the Education of Teachers in Science.

[CIT0022] Texas School for the Blind and Visually Impaired, 2007, ‘Evals Evaluating visually impaired students’, viewed 22 June 2015, from http://www.tsvbi.edu/curriculumapublications?catdi=3&id=1030:evlsevaluatingvisuallyimpaired-students

